# Pacing-Induced Regional Differences in Adenosine Receptors mRNA Expression in a Swine Model of Dilated Cardiomyopathy

**DOI:** 10.1371/journal.pone.0047011

**Published:** 2012-10-12

**Authors:** Silvia Del Ry, Manuela Cabiati, Vincenzo Lionetti, Giovanni D. Aquaro, Alessandro Martino, Letizia Mattii, Maria-Aurora Morales

**Affiliations:** 1 Institute of Clinical Physiology, CNR, Pisa, Italy; 2 Institute for Life Sciences, Laboratory of Medical Sciences, Scuola Superiore Sant'Anna, Pisa, Italy; 3 Fondazione CNR-Regione Toscana “G. Monasterio”, MRI Lab, Pisa, Italy; 4 Department of Biology, Genetics Division, University of Pisa, Pisa, Italy; 5 Department of Human Morphology and Applied Biology, Medical Histology and Embryology Section, University of Pisa, Pisa, Italy; University of Otago, New Zealand

## Abstract

The adenosinergic system is essential in the mediation of intrinsic protection and myocardial resistance to insult; it may be considered a cardioprotective molecule and adenosine receptors (ARs) represent potential therapeutic targets in the setting of heart failure (HF). The aim of the study was to test whether differences exist between mRNA expression of ARs in the anterior left ventricle (LV) wall (pacing site: PS) compared to the infero septal wall (opposite region: OS) in an experimental model of dilated cardiomyopathy. Cardiac tissue was collected from LV PS and OS of adult male minipigs with pacing-induced HF (n = 10) and from a control group (C, n = 4). ARs and TNF–α mRNA expression was measured by Real Time-PCR and the results were normalized with the three most stably expressed genes (GAPDH, HPRT1, TBP). Immunohistochemistry analysis was also performed. After 3 weeks of pacing higher levels of expression for each analyzed AR were observed in PS except for A_1_R (A_1_R: C = 0.6±0.2, PS = 0.1±0.04, OS = 0.04±0.01, p<0.0001 C vs. PS and OS respectively; A_2A_R: C = 1.04±0.59, PS = 2.62±0.79, OS = 2.99±0.79; A_2B_R: C = 1.2±0.1, PS = 5.59±2.3, OS = 1.59±0.46; A_3_R: C = 0.76±0.18, PS = 8.40±3.38, OS = 4.40±0.83). Significant contractile impairment and myocardial hypoperfusion were observed at PS after three weeks of pacing as compared to OS. TNF-α mRNA expression resulted similar in PS (6.3±2.4) and in OS (5.9±2.7) although higher than in control group (3.4±1.5). ARs expression was mainly detected in cardiomyocytes. This study provided new information on ARs local changes in the setting of LV dysfunction and on the role of these receptors in relation to pacing-induced abnormalities of myocardial perfusion and contraction. These results suggest a possible therapeutic role of adenosine in patients with HF and dyssynchronous LV contraction.

## Introduction

Adenosine derives from ATP degradation; it is considered to play an important role in adaptation to inadequate oxygen supply since it accumulates under hypoxic or ischemic conditions [1]–[3]. Adenosine concentration measurement is known to be technically difficult since it is rapidly degraded into inosine. Basal adenosine levels reported in myocardial interstitial fluid range from nano- to micromolar concentrations; maximal ATP-derived adenosine production might reach 100 nmol adenosine/(h.heart) in the case of oxygen shortage [4], [5]. The diverse cellular actions of adenosine are mediated by a family of adenosine receptors (ARs); four subtypes (A_1_R, A_2A_R, A_2B_R and A_3_) have been cloned and pharmacologically characterized [6], [7].

Evidence indicates that the adenosinergic system is essential in the mediation of intrinsic protection and in determining myocardial resistance to insult; therefore, adenosine may be considered as a potential cardioprotective molecule and ARs could represent potential therapeutic targets in the setting of heart failure (HF).

In a previous study, it was shown that AR subtypes are ubiquitously distributed in cardiac tissue and up-regulated in the left ventricle (LV) after 3 weeks of pacing in a model of pacing-induced HF in pigs [8].

A study carried out in our Institute demonstrated that high-frequency pacing of the LV free wall causes a dyssynchronous pattern of activation that contributes to progressive cardiac failure, with pronounced differences in regional contractility and flow but global abnormalities in myocardial metabolism [9].

Efficient LV chamber performance relies also on coordinated contraction of LV walls; a dyssynchronous pattern of activation - as observed during pacing in experimental models- contributes to progressive cardiac failure and can be related to what frequently reported in clinical practice in patients with conduction abnormalities who are more prone to develop left ventricular dysfunction [10].

The aim of the study was to test in this animal model of pacing-induced HF whether regional differences exist in ARs mRNA expression at pacing site (PS) as compared to the opposite wall (OS) and whether these differences possibly depend on regional abnormalities of myocardial contractility and perfusion.

## Materials and Methods

### Pacing protocol and tissue collection

Cardiac tissue was collected from two different sites of the LV of male adult minipigs (weight 35–40 Kg) with pacing-induced HF (n = 10).

Left intercostal thoracotomy was performed under general anesthesia and sterile conditions. An aortic catheter was surgically inserted in the descending aorta and an unipolar screw-type epicardial pacing lead was connected to the anterior LV free wall. A programmable pacemaker was implanted in a subcutaneous pocket. After surgery, the minipigs were allowed to fully recover for 10 days. Next, HF was induced by rapid LV pacing (180 beats/min) for 3 weeks. The cardiovascular function was monitored as previously described [9] and minipigs were considered to be in HF when the LV end-diastolic pressure was ≥20 mmHg and LV ejection fraction was <25%.

All animals underwent hemodynamic evaluation (heart rate, mean aortic pressure, left ventricular pressure and dP/dt_max_) at 10 days after surgery (baseline) and after 3 weeks of LV pacing. Global and regional myocardial contractility was assessed by magnetic resonance imaging (MRI, 1.5 Tesla) at baseline and at the end of the experimental protocol as previously described [9].

Finally the animals were deeply sedated with 3.3 mg/kg of propofol and sacrificed by injecting saturated KCl solution (10 ml). Cardiac tissue samples were collected from the anterior left ventricular wall, PS, and from the infero septal wall, (OS). The samples were immediately placed in ice-cold RNA*later* and stored at −80°C.

Cardiac tissue (left ventricular wall) was also collected from male adult minipigs without (n = 4) pacing-induced HF as control group (C).

These procedures were approved by the Italian Ministry of Health in accordance with the Italian law (DL-116,27 January 1992), which conforms to the Guide for the Care and Use of Laboratory Animals published by the US National Institute of Health (NIH publication n° 85-23, revised 1996).

### Hemodynamic recordings

The aortic catheter was attached to a strain-gauge transducer to measure arterial pressure [9].

LV pressure and dP/dtmax values were measured using a calibrated pressure catheter (Millar Instruments Inc, Houston, TX, USA) inserted percutaneously into the LV cavity under fluoroscopy guidance in animals lightly sedated with continuous infusion of midazolam (0.1 mg•kg^−1^•h iv) at spontaneous breathing [11]. All analogical signals were recorded and stored in a computer memory through an analog-digital interface (National Instruments), at a sampling rate of 250 Hz for subsequent analysis.

### Cardiac MRI

As previously described, cine-MRI images were acquired with a 1.5 Tesla MRI scanner (Signa Excite HD, GE Medical Systems,Waukesha, WI, USA) in sedated animals with continuous infusion of midazolam (0.1 mg•kg^−1^•h iv) at spontaneous heart rate. Global LV parameters (end-diastolic volume, end-systolic volume and ejection fraction) were analyzed in pacing-induced HF with a commercially available research software package (Mass Analysis, Leyden, The Netherlands) [9], [12]–[14].

Regional LV myocardial perfusion was evaluated with the first-pass MRI technique by the LV relative upslope of signal intensity (LVRUSI, myocardial upslope divided LV cavity upslope) for each myocardial segment, obtained after a bolus injection of gadolinium-diethylenetriamine pentaacetic acid (0.05 mmol/Kg at 4 ml/s of infusion velocity iv Gd-DTPA; Magnevist, Schering, Berlin, Germany) in a peripheral vein [9], [12].

To regionally assess the presence of tissue fibrosis, gadolinium-delayed contrast-enhanced images were acquired in two-dimensional segmented inversion recovery-prepared gradient echo sequence 10 min after administration of the contrast agent Gd-DTPA (0.2 mmol/kg iv) in short-axis views [9], [12]–[14].

### Molecular analysis

#### RNA extraction

Total RNA was extracted by acid guanidinium thiocyanate-phenol-chloroform method from tissue samples obtained from pig hearts with the Rneasy Midi kit (Qiagen S.p.A, Milano, Italy) as previously described [8], [15]–[17]. RNA concentration was determined spectrophotometrically (Biophotometer Eppendorf) at 260 nm. The ratio of readings at 260 nm and 280 nm (A_260_/A_280_) provided an estimate of the purity of RNA. The integrity and purity of total RNA was also detected by electrophoresis of samples on ethidium bromide agarose gels. Only the samples that showed clear and distinct 28S and 18S ribosomal RNA bands and had spectrophotometric OD 260/280 ratios of 1.9–2.1 were used. A known amount of total RNA (Ambion) was used as marker. The RNA samples were stored at −80°C for use in gene expression studies.

#### Real Time quantitative RT-PCR

Following DNAse treatment, first strand cDNA was synthesized with iScript cDNA Synthesis kit (Bio-rad, Hercules, California, USA) using about 1 µg of total RNA as template.

Real-time PCR was conducted on a 96-well iQ Cycler Real-Time PCR Detection System (Biorad, Hercues, CA, USA) in a 25 µl final volume, using 12.5 µl of iQ SYBR Green Supermix 2X (Biorad, Hercues, CA, USA) and 2 µl of cDNA as template. In general, the amplification protocol started with 95°C/3 min followed by 40 cycles of 95°C/15 s and 60°C/30 s. Annealing temperature varied depending on the primer pair used (Table 1). Two no template controls were used in each PCR run, while two inter-run calibrators were used to ensure the comparability of different PCR runs.

**Table 1:** Primers pairs.

Gene	Primers	Annealing T°	GeneBank
A_1_R	5′- ATCAGGTTACTTGGTTCT -3′ 5′- ATCAGGTTACTTGGTTCT -3′	57°	AY772411
A_2A_R	5′- GATCAGCCTCCGCCTCAACGGCCA -3′ 5′- TCAGGACACTCCTGCTCTGTCCTG -3′	60.5°	AY772412
A_2B_R	5′- TGGTGTACTTCAACTTCCTG -3′ 5′- GATCTTGGCGTAGATGGC -3′	60°	AY772413
A_3_R	5′- GGTGAAGTGCCAGAAGTTGTG -3′ 5′- AGCATAGACGATAGGGTTCATCAT -3′	60°	AY772414
TNF-α	5′- TGACCACCACCAAGAATT -3′ 5′- TGTTCTGAAGTATTCCGATTG -3′	60°	NM_214022
GAPDH	5′- TCGGAGTGAACGGATTTG -3′ 5′- CCTGGAAGATGGTGATGG -3′	59°	AF017079
HPRT1	5′- CCGAGGATTTGGAAAAGGT -3′ 5′- CTATTTCTGTTCAGTGCTTTGATGT -3′	60°	DQ178126
TBP	5′- GATGGACGTTCGGTTTAGG -3′ 5′- AGCAGCACAGTACGAGCAA -3′	59°	DQ178129

Primer pair used for housekeeping and target genes in Real Time experiments.

A_1_R = adenosine 1 receptor A_2A_R = adenosine 2A receptor, A_2B_R = adenosine 2B receptor, A_3_R = adenosine 3 receptor, TNF-α = tumor necrosis factor - α, GAPDH = Glyceraldehyde 3-phosphate dehydrogenase, HPRT1 = Hypoxanthine PhosphoRibosylTransferase 1, TBP = TATA-Binding protein.

A series of PCRs was performed on cDNAs of HF pigs (PS and OS respectively) using primer pairs for A_1_R, A_2A_R, A_2B_R, A_3_R and TNF-α. Primer pairs were designed with Primer Express Version 2.0 (Applied Biosystems) and details are given in Table 1. Specificity of each primer pair, i.e., absence of artifacts, multiple PCR products or primer-dimers, and PCR yield were checked by agarose-gel electrophoresis and by melting analysis. All reactions were performed in triplicate.

### Immunohistochemistry analysis

Myocardial tissue samples were paraffin-embedded, cut into 5-µm-thick sections, mounted serially on glass slides and treated for immunohistochemistry as previously described [18].

A_3_R expression was detected using a rabbit anti-human adenosine A_3_R antibody (ADORA3, MBL International Corporation, MA, USA) diluted 1∶100 in BSA-PBS. This antibody reacts with pig A_3_R on paraffined sections as specified by the supplier. For each selected specimens, at least three serial sections 1/10 were examined using semi-quantitative scale of immunoreactivity: no (−), low (+), medium (++) and high (+++) staining. Negative controls were obtained by incubating the specimens with BSA-PBS, omitting the primary antibody. Representative photomicrographs were taken by a DFC480 digital camera (Leica Microsystem, Cambridge, UK).

## Data Analysis

The geometric mean of the three most stably expressed genes (GAPDH, HPRT1,TBP), selected in previous studies [8], [19], was confirmed in this new set of experiments where C, PS and OS samples were considered as a single system which was used for normalization of each gene mRNA expression in the samples.

The relative quantification was performed by ΔΔCt method using iQ5 Software (BioRad, Hercules, CA, USA).

Differences between more than two independent groups were analyzed by Fisher's test after ANOVA. The results were expressed as mean ± SEM; p-value was considered significant when <0.05.

## Results

### Hemodynamic parameters

As shown in Table 2, after 3 weeks of pacing the animals showed an increased heart rate and a marked decrease in mean arterial pressure and LV end-systolic pressure.

**Table 2:** Hemodynamic parameters in controls and HF animals (after 21±2 days of pacing).

	Controls	HF	
**HR, beats/min**	92±7.2	108.5±8.64	p<0.0001
**LVEDP, mmHg**	7.4±0.85	21.5±3.26	p = 0.0002
**MAP, mmHg**	101.1±10.7	81.8±10.8	p<0.0001
**LVDP/dmax, mmHg/s**	2064.2±115.9	1536.1±151.1	p<0.0001
**LVESP, mmHg/s**	116.1±12.4	95.0±8.25	p<0.0001

HR: heart rate, LVEDP: left ventricular end-diastolic pressure, MAP: mean arterial pressure, LVESP: left ventricular systolic pressure.

In all animals LV end diastolic pressure was at least 20 mmHg which was considered an index of severe, although not end-stage, HF.

### Cardiac MRI

At the end of the experimental protocol global LV ejection fraction was significantly reduced (35.9±3 vs. 76±2%, p<0.05). In particular, LV end-systolic wall thickening (LVESWT), an index of regional contractile function, was significantly lower in the PS than in the OS (5.31±2.08 vs. 45.04±4.18, p<0.001), indicating a severe regional pacing-induced contractile impairment. The LVRUSI was more reduced in the PS than in the OS (9.1±1 vs. 14.6±1.2%, p<0.05), suggesting a significant myocardial hypoperfusion in the abnormally contracting area. No changes between LV PS and OS were reported for gadolinium-delayed enhancement, suggesting lack of significant tissue fibrosis.

### ARs, and TNF-α mRNA expression in PS and OS

**Figure 1:**
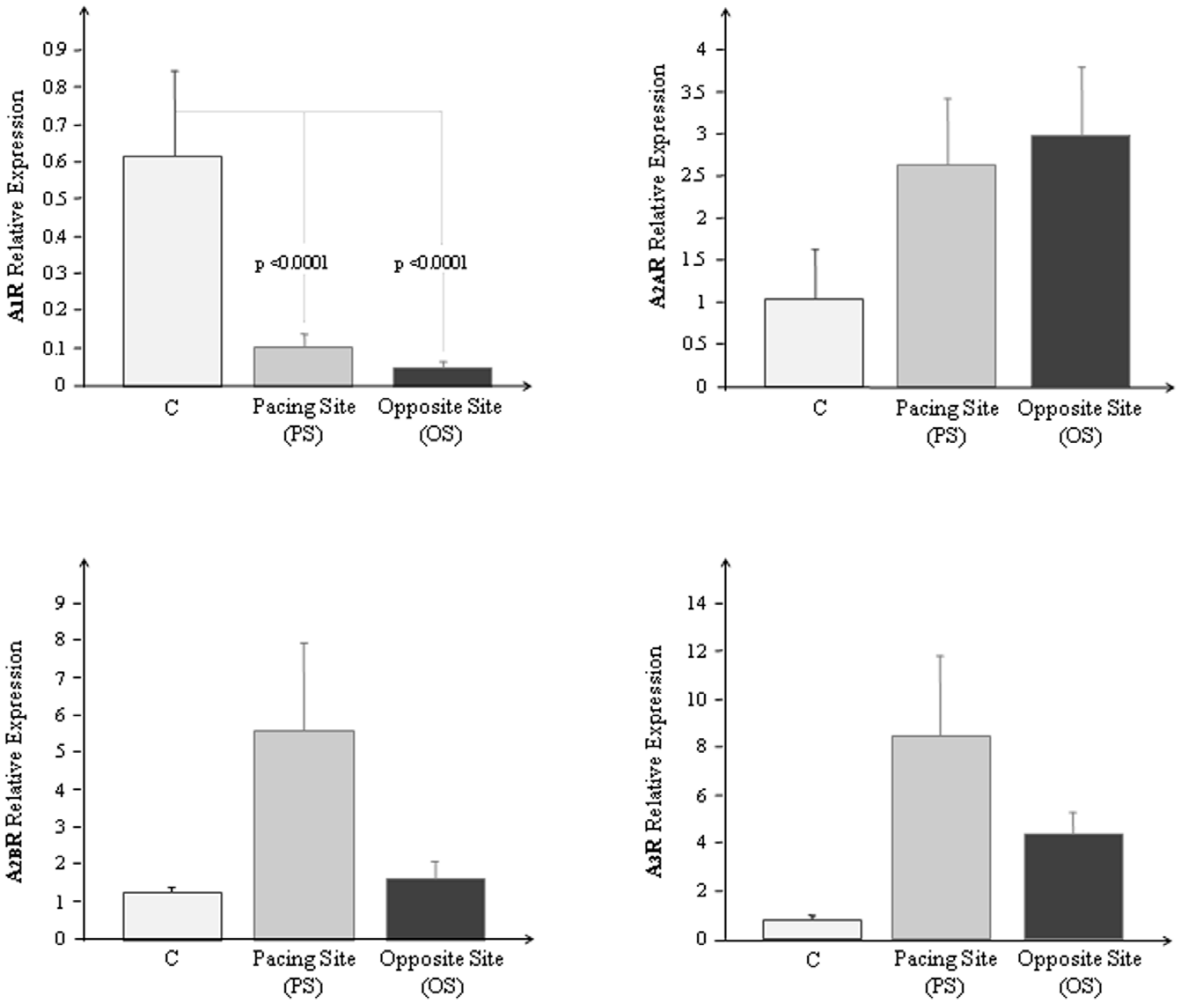
Adenosine receptors mRNA expression in left ventricle of normal and HF minipigs. **a**) A_1_R, **b**) A_2A_R, **c**) A_2B_R and **d**) A_3_R mRNA expression measured by Real Time PCR in the left ventricle of controls (C) (white bars), in the anterior left ventricular wall (PS) (grey bars) and in the opposite site (OS) (black bars) of HF minipigs. The result were expressed as mean ± SEM.

In Fig. 1 the mean values obtained for A_1_R, A_2A_R, A_2B_R and A_3_R mRNA expression at PS, OS in HF animals and in C, are reported.

After 3 weeks of pacing higher levels of expression for each ARs were observed in PS except for A_1_R; for this subtype a significant mRNA reduction was found both in PS and in OS (p<0.0001, respectively).

Higher levels of A_1_R, A_2B_R and A_3_R although not statistically significant were observed in PS as compared to OS. A_2A_R were almost comparable in the two sites.

TNF-α mRNA expression resulted similar in PS (6.3±2.4) and in OS (5.9±2.7) in HF minipigs, although higher than in the control group (3.4±1.5).

A very significant correlation was also found between overall A_2B_R and A_3_ (r = 0.88, p<0.0001).

### Immunohistochemistry

To confirm mRNA expression of the AR receptors, myocardial A_3_ expression was assessed by immunohistochemistry.

**Figure 2:**
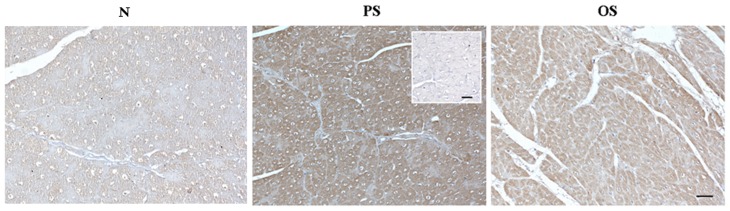
A_3_R immunohistochemical expression. Immunohistochemical staining of A_3_R in the left ventricle of controls (C), in the anterior left ventricular wall pacing site (PS) and in the opposite site (OS) of HF minipigs. Negative control was inserted in the right side of PS image. Scale bar: 50 µm.

As showed in Fig. 2 A_3_R immunohistochemical expression reaction (brown) was greater in PS (+++) than OS (++) and C (+) while the negative control was not immunoreactive (−).

## Discussion

In a previous study [8] performed in the same experimental model, an up-regulation of ARs in the left ventricle was found, as compared to controls, after 3 weeks of sustained high frequency LV pacing. While 7-5-2 fold increase was observed for A_2B_R, A_3_R and A_2A_R, with respect to the control group, A_1_R showed a slight increase; however, whether the myocardial expression of ARs would relate to the regional distribution of LV contractile dysfunction was not assessed.

In the current investigation the ARs mRNA expression was evaluated in the PS and in the OS of an animal model of non-ischemic dilated cardiomyopathy induced by LV pacing at high frequency as well as in a group of healthy animals. ARs expression in the regions of interest of the LV myocardium was assessed and a receptor up-regulation in the PS was found compared to OS. The trend of ARs mRNA expression previouslydetermined [8] in control group vs. HF animals in the left ventricle was confirmed except for A_1_R mRNA which resulted significantly reduced both in PS and in OS, probably due to the larger number of studied animals.

ARs are known to be activated by interstitial physiological or supraphysiological concentration levels of adenosine [5]. As observed in different models exposed to varying concentrations of an A_1_R agonist, a rapid internalization of A_1_R is possible, leading to receptor desensitization [20], [21].

The lower level of A_1_R observed in this study both in PS and OS after HF induction could be due to a desensitization of A_1_R caused by higher adenosine concentration. These results are in line with a previous study in which agonist-induced cell surface A_1_R aggregation with subsequent receptor desensitization and internalization was demonstrated [20].

The reduction in pump function after high frequency ventricular pacing is due not only to tachycardia but also to the abnormal electrical activation which determines the loss of the normal apex-to-base sequence of activation, as seen during normal sinus rhythm, leading to an asyncronous contraction of the different parts of the left ventricular myocardium [22]–[24].

After a few weeks of continuous high-frequency pacing, myocardial perfusion was markedly impaired, especially in regions with a more pronounced contractile derangement.

The semi-quantitative MRI assessment showed higher perfusion levels in the OS relative to the PS while no differences could be found in terms of tissue fibrosis between LV PS and OS by gadolinium-delayed enhancement techniques.

Our findings provide the first evidence of regional differences in ARs mRNA expression in a dysfunctioning heart with LV asynchronous contraction.

Although the lack of statistically significant values may limit the strength of the results, the trend observed at the two LV walls and, mostly, the influence of pacing on local perfusion and contractility, together with the differences with controls, may provide important information on local receptor expression in this widely used experimental model.

The long-term asynchrony and impairment in LV contractile performance seem to exert a positive feedback on the transcription of genes coding for myocardial adenosine receptors not associated with collagen synthesis.

Adenosine protects the heart during ischemia via the activation of A_1_R and A_3_R [25], increasing coronary blood flow via the activation of A_2A_R, improving contractile performance and inhibiting cytokine production and inflammatory response [26], [27].

In this study A_1_R, A_2B_R and A_3_R mRNA expression resulted higher in the PS than in the OS; the lower myocardial levels of ARs in OS could be in tune with the presence of better myocardial contraction and perfusion in this region as compared to PS.

These results are confirmed by immunohistochemistry and are in agreement with a previous study where higher ATP consumption and better mechanical activity in the OS of a failing heart was observed [28].

The higher TNF-α levels found in HF animals as compared with controls support the hypothesis of the interaction between adenosine and TNF-α in the initiation, maintenance and amplification of the inflammatory response [8].

This model, devoid of possible confounding effects of pharmacological therapy, allowed the evaluation of alterations of ARs in the setting of LV dysfunction and provided new data on the role of these receptors in relation to pacing-induced abnormalities in myocardial perfusion and contraction. These results suggest a possible therapeutic role of adenosine in patients with heart failure and dyssynchronous contraction. Chronic dyssynchrony profoundly affects [10], [29] and accelerates the progression of heart failure and represents the rationale of resynchronization therapy to reduce the burden of incoordinate LV contraction [30]. It could be hypothesized that in HF patients with normal coronary arteries and LV dyssynchrony, adenosine might represent a possible therapeutic approach to slow down or even revert the development of heart failure.
